# A novel protein descriptor for the prediction of drug binding sites

**DOI:** 10.1186/s12859-019-3058-0

**Published:** 2019-09-18

**Authors:** Mingjian Jiang, Zhen Li, Yujie Bian, Zhiqiang Wei

**Affiliations:** 1 Department of Computer Science and Technology, Ocean University of China, 238 Songling Road, Qingdao, 266100 China; 2 Pilot National Laboratory for Marine Science and Technology (Qingdao), 1 Wenhai Road Aoshanwei, Qingdao, 266237 China

**Keywords:** Binding sites prediction, Deep learning, Molecule descriptor, Protein pockets

## Abstract

**Background:**

Binding sites are the pockets of proteins that can bind drugs; the discovery of these pockets is a critical step in drug design. With the help of computers, protein pockets prediction can save manpower and financial resources.

**Results:**

In this paper, a novel protein descriptor for the prediction of binding sites is proposed. Information on non-bonded interactions in the three-dimensional structure of a protein is captured by a combination of geometry-based and energy-based methods. Moreover, due to the rapid development of deep learning, all binding features are extracted to generate three-dimensional grids that are fed into a convolution neural network. Two datasets were introduced into the experiment. The sc-PDB dataset was used for descriptor extraction and binding site prediction, and the PDBbind dataset was used only for testing and verification of the generalization of the method. The comparison with previous methods shows that the proposed descriptor is effective in predicting the binding sites.

**Conclusions:**

A new protein descriptor is proposed for the prediction of the drug binding sites of proteins. This method combines the three-dimensional structure of a protein and non-bonded interactions with small molecules to involve important factors influencing the formation of binding site. Analysis of the experiments indicates that the descriptor is robust for site prediction.

**Electronic supplementary material:**

The online version of this article (10.1186/s12859-019-3058-0) contains supplementary material, which is available to authorized users.

## Background

A new drug needs to go through multiple stages before entering the market, including the discovery of new drug compounds (called lead compounds), clinical research, marketing, and tracking. The discovery of lead compounds is the most important and time-consuming of these stages. The traditional method involves chemical experiments in the laboratory and reactions of various small molecules with a target protein; then, the binding site for a small molecule in the complex is used as a protein pocket. This approach requires considerable manpower and time. Currently, computer-aided drug design has gradually become a new trend because of the skyrocketing costs of drug development [1]. Virtual screening of small molecules that can bind to a target protein is a common step in computer-aided drug design. This process can identify a small subset for experimental testing [2]. The location of the binding sites is critical for screening. At present, the structures of numerous protein complexes have been obtained by the experimental methods and are collected in a number of databases [3–5]. However, numerous proteins have no information about their binding sites, thus limiting the speed of drug design. Hence, finding an automatic site prediction method is crucial in drug design.

The computational methods of site prediction can be divided into sequence-based, energy-based and geometry-based methods. Initially, the binding sites are usually predicted by using the three-dimensional geometric structure of a protein by searching for the cavities and pockets; this is called the geometry-based method. Laskowski [6] proposed a method called Surfnet to predict the potential pockets of a protein by filling the spheres between the atom pairs of a protein and a small molecule to find a surface gap or a cavity. Le Guilloux et al. [7] used the spheres to find the pockets; however these authors used the Veno partitioning algorithm called Fpocket to filter out spheres within a threshold, which are the cavities on the surface of the protein. In addition, certain geometry-based methods utilize grids, such as LIGSITE [8] and LIGSITE ^*csc*^ [9], which look for the proposed protein-solvent-protein events and surface-solvent-surface events by constructing the grids. Then, the pockets are predicted by the grid values. bSiteFinder [10] also used the structure of the proteins to find the pockets by looking for the proteins with the same structure as target protein; the known binding sites are regarded as references to recognize the sites. Certain other methods, such as CAST [11], PASS [12], and PocketPicker [13], used the geometric methods to explore the pockets or cavities. Geometry-based methods identify pockets by looking for cavities on the surface of the proteins. The methods work well when looking for a rigid binding pocket but are not adapted to find flexible binding pockets, thus limiting the abilities of the binding site predictions.

The energy-based method used in flexible docking estimates the energy of each position of a protein through a probe and predicts the binding site by the distribution of the energy values. In 1984, Goodford [14] used probes to calculate the van der Waals forces, hydrogen bond potentials, and electric potentials for various grid points of the proteins, and predicted the binding sites based on the calculated energy values. Laurie proposed a method called Q-SiteFinder [15], which used the −*CH*_3_ probe to calculate the non-bonded interaction; a clustering algorithm was implemented to cluster the final energy distribution to predict the potential pockets. PocketFinder [16] used a transformation of the Lennard-Jones potential calculated from a three-dimensional protein structure and did not require any information about a potential ligand molecule. There are numerous models for the calculation of non-bonded interactions that are usually called scoring functions, such as AutoDock Vina scoring function [17] and Vardo [18]. Moreover, Bitencourt-Ferreira et al. developed a model to predict Gibbs free energy of binding for the protein-ligand complexes [19] using the machine learning methods available in the SAnDReS program [20]. The Lennard-Jones potential [21] is the most common and simple energy calculation method.

Sequence-based methods typically focus on the sequence of a protein, and the results of site prediction include the residues with binding activity. For example, Schelling et al. proposed a method to predict active residues from the evolutionary couplings and sequence variation [22]. Kumar proposed a site prediction method that used simplified amino acid alphabets as features to feed a random forest model; however, this method is only suitable for predicting the sites of metal ions. Similarly, ZincBinder [23] utilized a support vector machine, which can predict a zinc metal-binding site in a protein using the sequence profile information. Haberal et al. proposed a deep convolutional neural network architecture called DeepMBS to predict the protein metal binding sites [24]. The authors encoded a protein residue by a set of numeric features and a window around the current residue was used to transform the corresponding subsequence into a vector of concatenated PAM (Point Accepted Mutation) representations of amino acids in the chain. Furthermore, Han et al. [25] developed a sequence-based method for predicting protein functional sites based on the assumption that proteins sharing similar structure and sequence tend to have similar functional sites located at the same positions on the protein’ surface. To avoid the over-fitting problem, Chen el at. [26] proposed a dynamic ensemble approach that constructs several balanced data sets, a random forest classifier was trained for each of the data sets. Then, a subset of classifiers was dynamically selected according to the similarity between the target protein and the proteins in the training set to get the final predictions. COFACTOR [27] predicts binding sites by identifying the template proteins of similar folds and functional sites from the protein residues and atoms. Additionally, CASTp [28] can be used to investigate surface features, functional regions and specific roles of the key residues of the proteins.

In addition, there are certain algorithms that combine some of the above methods, such as ConCavity [29] and FINDSITE [30], which are integrated into COACH [31] and can achieve good results. It has been suggested that combing multiple methods may help to improve the performance of the descriptors for prediction of the binding sites.

In recent years, new deep learning techniques have been used in drug discovery and development, opening a new door to computational decision making in pharmaceutical science [32].For example, DeepAffinity [33] was proposed to predict the compound-protein affinities with unified recurrent and convolutional neural networks. Zheng et al. summarized the use of text mining applications in drug discovery [34]. Numerous sequence-based methods utilized the deep learning model, which extracted the features from the protein sequences and predicted the binding sites using the deep-learning architecture, including MusiteDeep [35], DeepMBS [24] and CNNsite [36]. Similarly, Cai et al. [37] used the machine learning methodology to mine the information from physicochemical properties (PCP) data concerning protein sequences; Efficient Bayesian Multivariate Classifier (EBMC), Support Vector Machine (SVM) and Logistic Regression (LR) are superior for prediction of the ubiquitination sites. In addition, the deep learning methods are applied in the prediction of protein binding sites. Jimenez [38] et al. proposed a novel method called DeepSite to detect pockets, which constructs a three-dimensional structure of the proteins according to atomic types. The 8-channel feature was extracted as an input to perform training on a convolutional neural network to ultimately predict the positions of the pockets. However, DeepSite only considers the L-J potential [21] energy of the atoms.

Construction of a robust protein descriptor is a critical step in the prediction of binding sites using machine learning methods and especially deep learning methods. Appropriate protein descriptor needs to reflect the *factors that influence the formation of the binding sites in a protein* and must be suitable for the neural network input. Similar to DeepSite, we have built a grid-based multi-channel descriptor that can more accurately describe a protein. The experimental results show that a model built with this descriptor is more accurate.

## Results

In this work, a multi-channel molecular descriptor for the prediction of protein drug binding sites is proposed, and appropriate super parameters are obtained in the experiments. The descriptor is more accurate than other methods of site prediction.

### Evaluation

In certain pocket prediction methods, such as PocketPicker [13], the hit rate is used to evaluate the performance. More specifically, if a predicted site is within 4Å of any atom of a ligand, the prediction can be regarded as a hit of the actual site. This study uses a more accurate metric, which is the distance from the center of the prediction binding site to the center of the actual site. In addition, certain proteins have more than a single predicted site, and only the top three score pockets are used for performance comparison in these cases. In other words, if a protein has more than three predicted sites, we identify three predicted binding sites with the highest scores, and the predicted site closest to the actual site is selected from the three sites as the site prediction and is used for evaluation; this approach is called as Top3 prediction (similar to Top5 prediction). In the experiments, sc-PDB [4] was used for performance comparison and selection of the hyper parameters. Random proteins are selected as a training set, a validation set and a test set. PDBbind [5] was used to verify the generalization of the model trained using sc-PDB. Both databases are public and accessible through their websites.

### Experiment with various channels

**Fig. 1:**
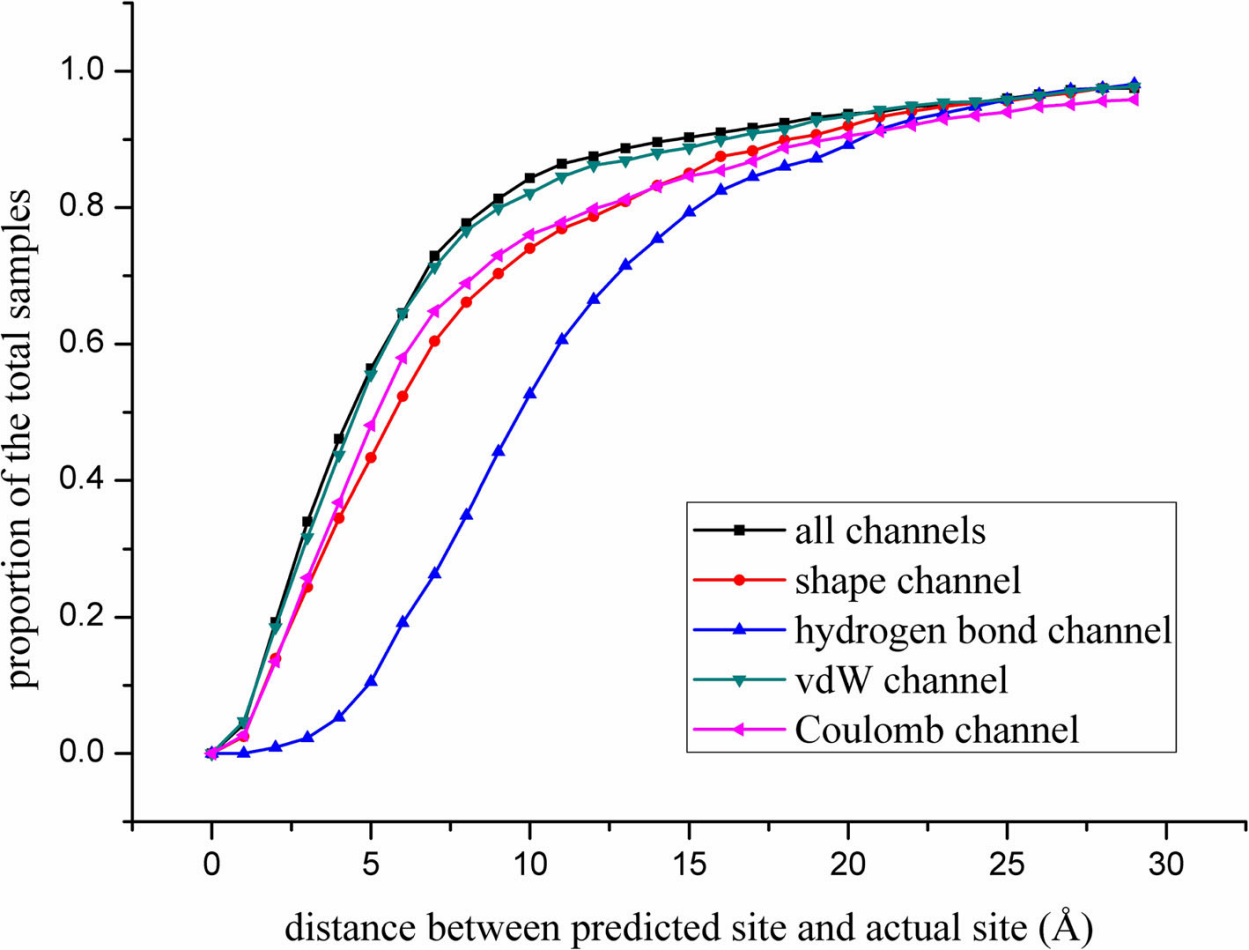
Performance comparison of different channels

To better identify the contribution of various channel factors to the prediction of protein binding sites, the performance of various channels was tested, including the full-channel model and other four single-channel models (shape, hydrogen bond, vdW force and Coulomb force channels). A total of 3000 proteins were randomly selected for training; 1000 proteins were selected for validation and 1000 proteins were selected for testing. The data set used is available in the Additional file 1, and the experimental results of various channels are shown in Fig. 1.

The data of Fig. 1 indicate that when all four channels of the descriptor are included, the prediction accuracy is the highest, because the factors influencing the formation of the binding sites are fully taken into account. In the case of the other four single-channel models, the vdW force channel model has the best experimental performance. The van der Waals channel may play an important role in the prediction of the binding site.

### Experiment with various DBSCAN parameters

**Fig. 2:**
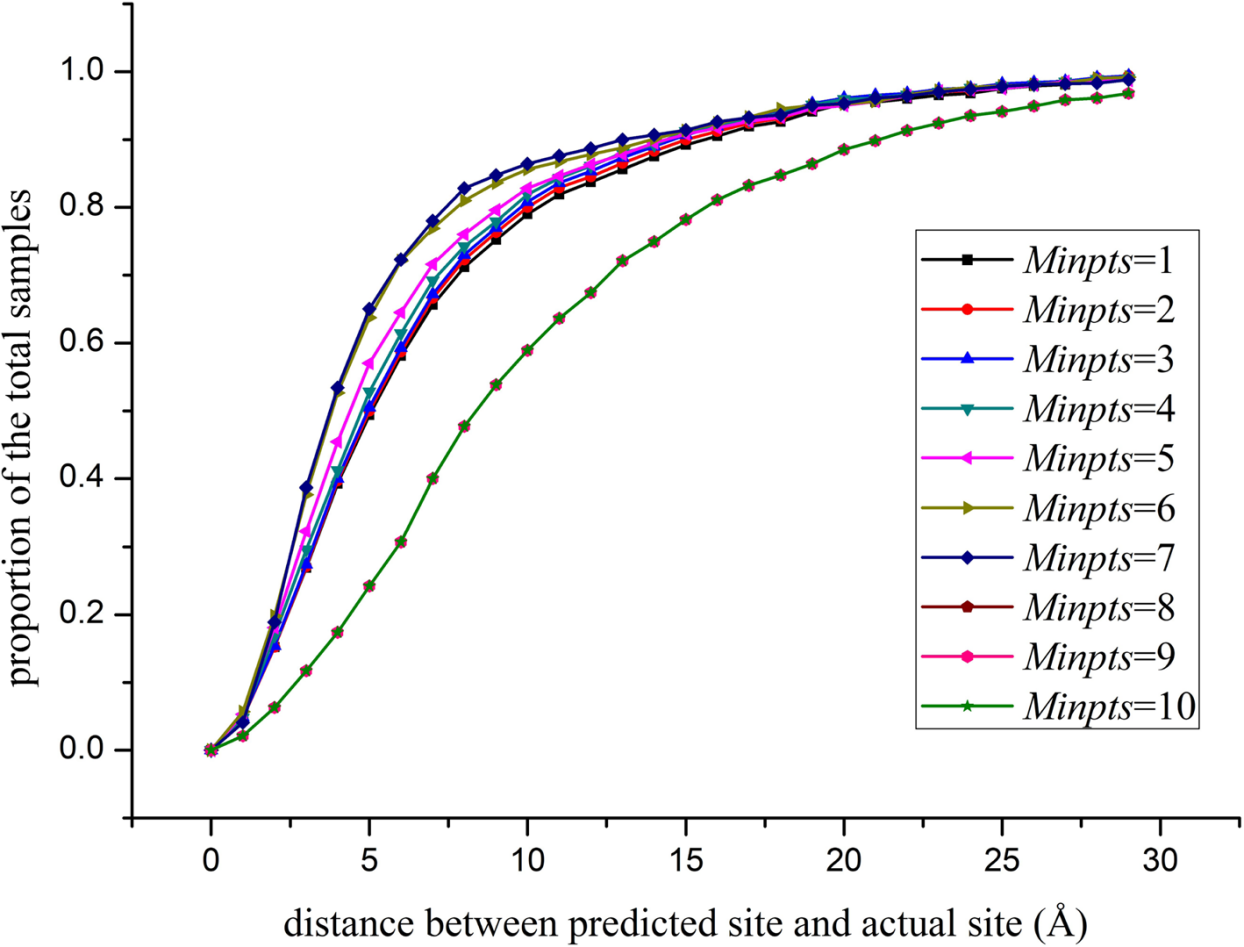
Comparison of different *Minpts* values for DBSCAN

In the process of binding sites prediction, all sampling blocks that exceed the threshold need to be clustered by the DBSCAN (Density-Based Spatial Clustering of Applications with Noise [39]) algorithm. We set *step*=4 for the sampling step so that the size of *Eps* is set as *step*+1=5 for DBSCAN to ensure that at least two sampling blocks close to each other are grouped. The *Minpts* parameters were set as *Minpts*=*i*(*i*=1,2,3…,10) to observe the clustering performance; 5000 randomly selected proteins (3000 for training, 1000 for validation and 1000 for testing; the data set used is available in the Additional file 2) were used for the experiment. The results are shown in Fig. 2.

The data of Fig. 2 indicate that when *Minpts* is set to 7, the performance of the clustering is the best. An increase in *Minpts* is associated with gradual worsening of the performance. The value reflects the lowest number of the sampling blocks clustered into a group, and a large value means that more blocks should be clustered into a group. High number sampling blocks need to be clustered into a class at increased values, which ultimately leads to unsatisfactory results. A smaller value means that the number of the sampling blocks in each class can be very small resulting in too many classifications and inaccurate predictions.

### Experiment on sc-PDB using various methods

**Fig. 3:**
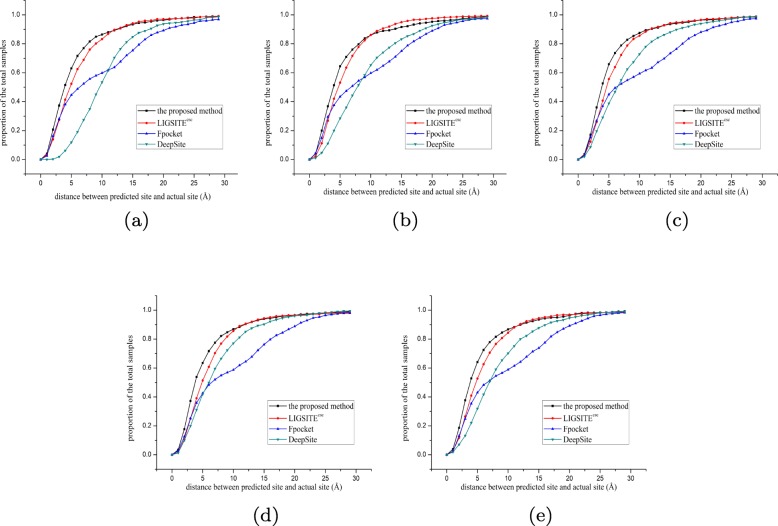
5-fold crossover experiment for Top3 prediction. **a** fold1 **b** fold2 **c** fold3 **d** fold4 **e** fold5

**Fig. 4:**
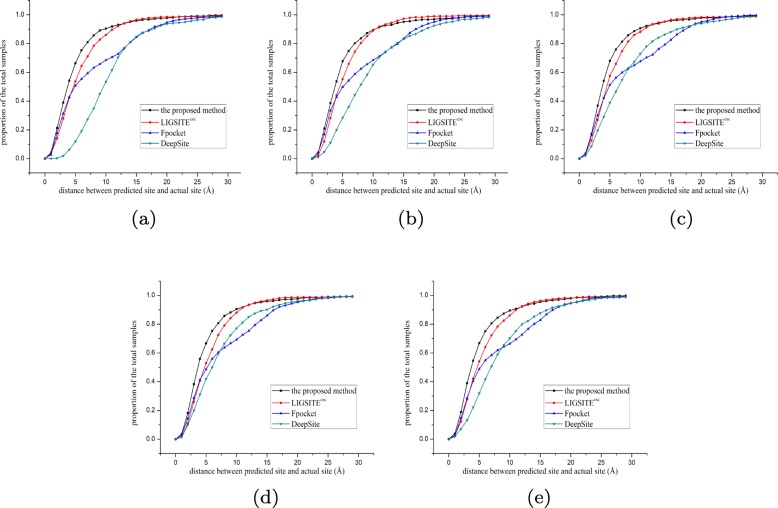
5-fold crossover experiment for Top5 prediction. **a** fold1 **b** fold2 **c** fold3 **d** fold4 **e** fold5

**Fig. 5:**
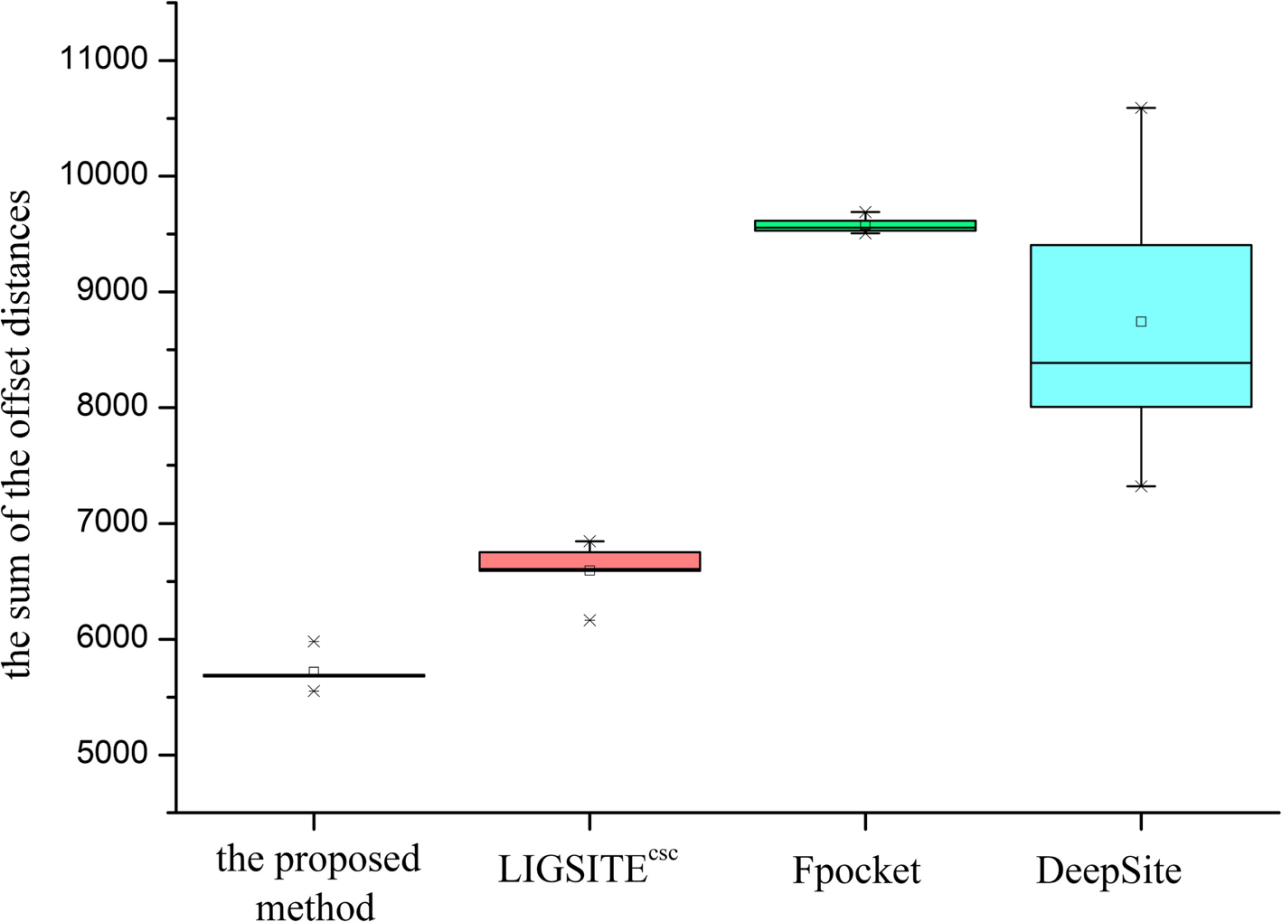
Error sum of different methods for Top3 predictions

**Fig. 6:**
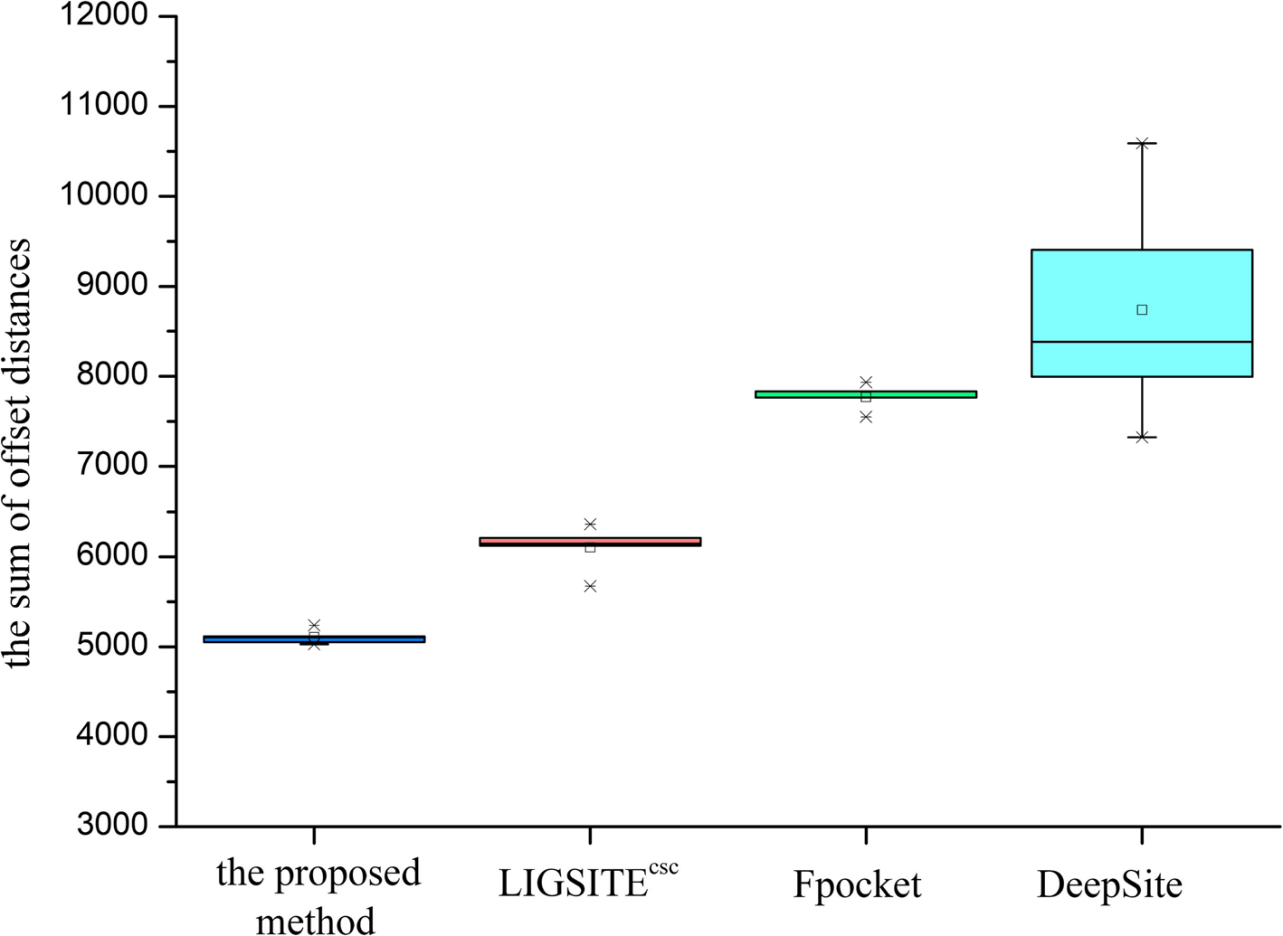
Error sum of different methods for Top5 predictions

The performances of various methods (the proposed method, DeepSite, Fpocket, and LIGSITE ^*CSC*^) were compared. For the training of the proposed method and DeepSite, the same training and validation sets were used. For more accurate analysis, 5000 randomly selected proteins (available in the Additional file 3) were used in a 5-fold crossover experiment; 4000 proteins (3000 for training and 1000 for validation) were used for training and 1000 proteins were used for testing in each fold. In addition, Fpocket and LIGSITE ^*CSC*^ may have more than five site predictions; hence, the Top5 prediction results were also analyzed. Figures 3 and 4 show the proportion of various offsets (the distance between the predicted site and the actual site) for various methods. Figures 5 and 6 display the sum of errors (the sum of the 1000 test protein offsets) predicted by various methods; Tables 1 and 2 show the number of predictions closest to the actual site in the 1000 test proteins per fold for various methods.

**Table 1:** The number of the closest predictions for Top3 prediction

method	fold1	fold2	fold3	fold4	fold5
the proposed method	367	344	347	377	410
LIGSITE ^*CSC*^	279	239	238	194	233
Fpocket	284	299	259	233	234
DeepSite	70	118	156	196	123
all	1000	1000	1000	1000	1000

**Table 2:** The number of the closest predictions for Top5 prediction

method	fold1	fold2	fold3	fold4	fold5
the proposed method	420	371	346	377	407
LIGSITE ^*CSC*^	225	220	218	177	212
Fpocket	304	320	288	266	268
DeepSite	51	89	148	180	113
all	1000	1000	1000	1000	1000

In the fivefold cross experiments of Top3 and Top5 predictions, the proposed method has a higher hit accuracy, especially within 5 Å. The data shown in Fig. 5 for Top3 prediction indicate that the sum of the offset distances of the proposed method for 1000 proteins is approximately 6000 Å; hence, the average distance for each protein is approximately 6 Å. The results are better than that in the other three methods. In the case of the Top5 prediction (Fig. 6), the average distance for each protein is 5.5 Å, which is also better than that in the other three methods. Based on the number of the Top3 and Top5 predictions predicted by various methods that hit the prediction closest to the actual site, the proposed method predicted closer binding sites for more proteins in the test set per fold. Therefore, the comprehensive comparison shows that the proposed method has a good prediction performance, which also implies that the proposed descriptor is very robust and accurate in predicting the protein binding sites. On the one hand, the cavity can be screened by the proposed method based on the shape of the protein (channel 1). On the other hand, a combination with the energy-based method enables detection and localization of the energy distribution (channel 2,3, and 4) of the probe. The binding factors of a protein and a drug molecule are comprehensively considered thus resulting in a higher hit rate of the site by the proposed method.

At the same time, the number of binding sites predicted by each method was counted, and this value is shown in Table 3. Using DBSCAN, potential predictive pockets of a protein are clustered to compress the number of predicted pockets for more accurate pocket positioning.

**Table 3:** The average number of predictions for a protein using various methods

method	fold1	fold2	fold3	fold4	fold5	mean	std
the proposed method	2.526	2.65	2.861	2.636	2.606	2.6558	0.11123021
LIGSITE ^*csc*^	-	-	-	-	-	-	-
Fpocket	27.806	27.671	27.88	27.439	27.065	27.5722	0.29467229
DeepSite	1.181	1.449	2.018	2.054	1.6	1.6604	0.3349511

### Experiment on PDBbind

**Fig. 7:**
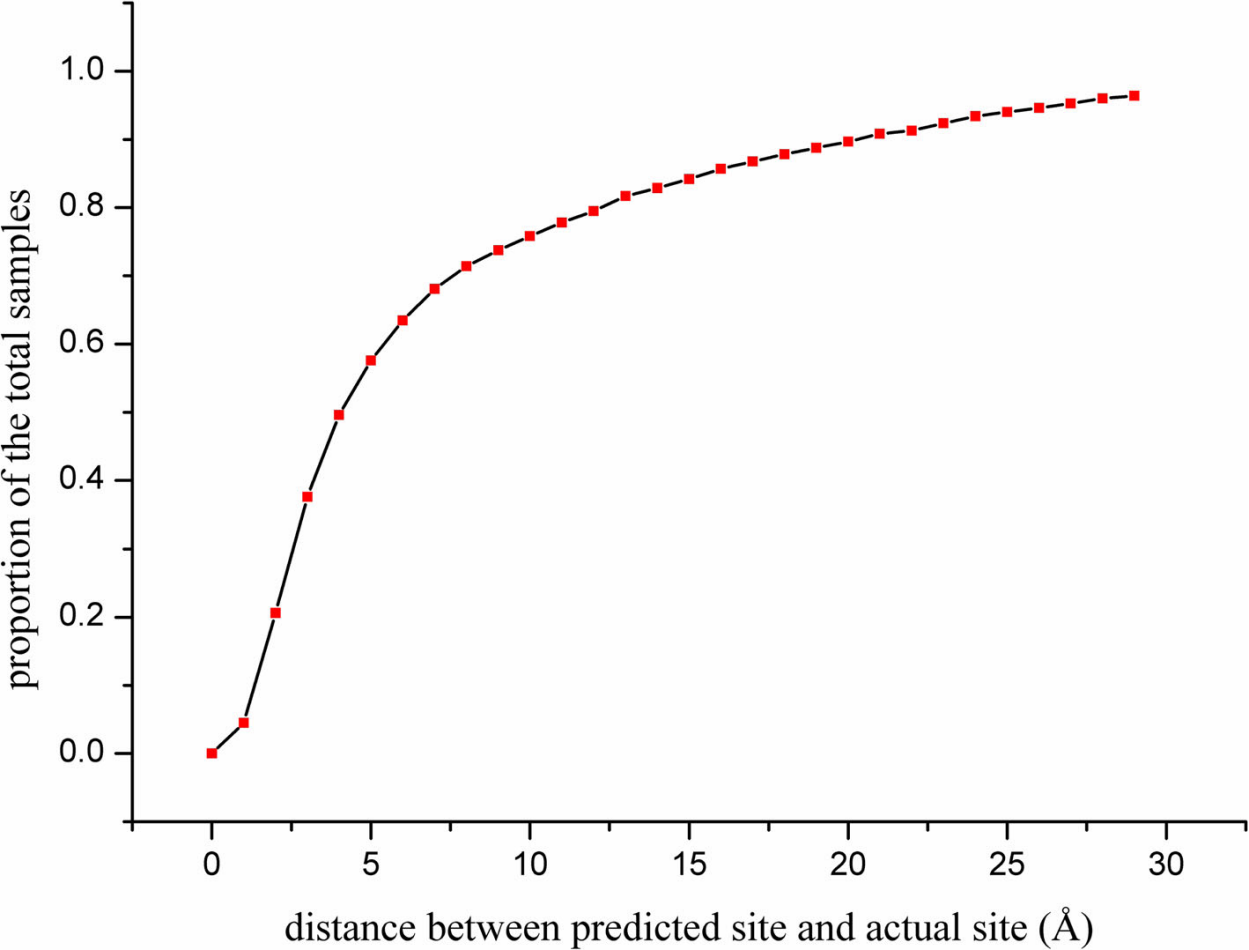
Generalization effect on PDBbind of the model trained using scpdb dataset

If the model trained in a dataset can be properly applied to other datasets, we can avoid retraining a new model with new data required for prediction of the binding sites in other datasets or in new unknown proteins. To verify the generalization of our model, the model was trained on 4000 proteins (3000 for training and 1000 for validation) in the sc-PDB dataset and subsequently used to predict the binding sites of proteins in the PDBbind dataset. We removed the proteins that appeared in the training and validation sets of sc-PDB and finally randomly select 1000 new proteins in the PDBbind dataset; the dataset used in this experiment is available in the Additional file 4. The prediction performance of the model used on these proteins is shown in Fig. 7. The results indicate that the test of the model on the PDBbind dataset still has a high probability (nearly 60%) of hit rate within 5 Å for Top3 prediction, although the performance is not as good as that in the case of sc-PDB. The reason for the decreased accuracy is that sc-PDB and PDBbind are different in the secondary processing of the original proteins. Nevertheless, the models trained on various datasets have better generalization, which is of great help for pocket prediction in new proteins. The average number of the predicted pockets is 2.72.

## Discussion

Computer-aided drug design has become popular, and the utilization of deep learning to predict the drug binding sites has gradually become a focus because of full use of the existing data resources and full exploitation of the information contained in the data.

Construction of appropriate protein descriptor is the first and foremost problem to be solved while using deep learning, especially the convolutional neural network, to predict the drug binding sites in a protein. To address this problem, a four-channel grid protein descriptor was constructed by analyzing the factors influencing the formation of the binding sites.

The constructed protein descriptor consists of four channels. First, shape is an important factor influencing the formation of the binding sites. A simplified version of LIGSITE is constructed for the first channel to describe the cavities on the protein surface. Second, hydrogen bonds and van der Waals forces play important roles in the binding between a protein and a ligand, and the L-J potential is used to construct the hydrogen bond energy grid and the van der Waals force grid. In addition, the Coulomb force is important for site prediction. The partial charges of the protein atoms are used to construct the grid of this channel.

A refined convolutional neural network is introduced for training. The experiments show that the constructed multi-channel grid descriptor is more accurate and robust in predicting the protein binding sites.

## Conclusions

Computer-aided drug design can accelerate drug development, and the prediction of the binding sites is a crucial step in computer-aided drug design. After analyzing the problems and drawbacks of the geometry-based and energy-based methods, we combined these two methods to construct a protein descriptor, which is adapted to deep learning specifically for the detection of the protein drug binding sites. The three-dimensional structure of the protein and the non-bonded interactions that influence the formation of the binding sites are introduced to construct the descriptor. Experiments were conducted to compare the accuracy of the proposed method with the previous methods. The experimental results show that the proposed descriptor is more accurate in predicting the binding sites. We have carried out the generalization experiment on other datasets using the trained model, and the results show the generalization ability of the descriptor. It is possible that other factors may influence the formation of the binding sites, including hydrophobicity etc. If these factors can be described in other channels of the grid voxel, the accuracy may be further improved. Our future work will focus on improving the descriptor based on these factors.

## Methods

### Construction of the descriptor

It is obvious that the geometry-based approaches take protein shape into consideration and look for gaps or cavities on the surface of a protein. The energy-based methods take into account the potential energy factors influencing the formation of the binding sites, such as hydrogen bonds, van der Waals forces and electric potential energy. Thus, a combination of the two methods may have better performance. In addition, application of the deep learning method for protein binding site prediction requires that the protein descriptor is suited for the model input. Fortunately, grid-based approaches can solve this problem, since the grid voxels of the proteins are similar to the pixels of images, and the multi-attribute channel grid is analogous to the RGB channel image. Importantly, the conventional neural network used in image processing is also suitable for the protein multi-channel grid. However, the input of the network needs to be changed from a 2D image to a 3D grid. Based on these considerations, various factors influencing the formation of a protein pocket are introduced in the proposed descriptor. Geometry and energy-based methods are combined to construct a multi-channel protein descriptor utilizing the grid voxel. The constructed protein descriptor is a 4-channel grid, which consists of a shape channel, a van der Waals potential energy channel, a hydrogen bond potential energy channel, and an electric potential energy channel.

Initially, a bounding box of a protein is constructed and an 8Å buffer is added to the surroundings of the box. The protein bounding box is subsequently divided into a grid of 1Å ×1Å ×1Å voxels. The final grid is processed to obtain four channels of the descriptor as described below.

#### The shape of the protein(channel 1)

**Fig. 8:**
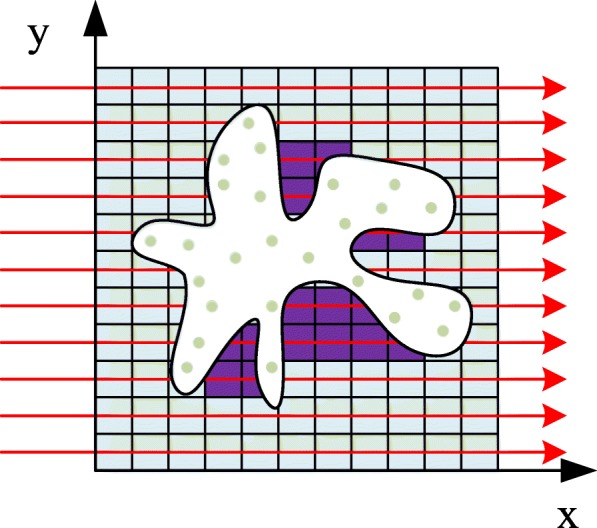
A slightly modified version of LIGSITE. The voxels represent the solvent, the green dots are the protein atoms, and the white area is the protein contour. The red lines are the scanning lines in the x direction with a step of 1Å. When a scanning line experiences a protein-solvent-protein event, the voxel contained in the intermediate solvent undergoes a PSP event indicated by the purple voxels. In three-dimensional case, proteins are scanned in seven directions including x,y,z and four diagonal directions

Structure is an important factor influencing pocket formation, and it is the focus of the original studies to the prediction of binding sites. In this channel, the LIGSITE method is slightly improved. A protein is mapped into a 3D grid, and a grid voxel becomes a part of the protein if it is within an atomic van der Waals radius of any protein atom; otherwise, it is considered to belong to the solvent. Then, the grid is scanned in x, y, z axes and four cubic diagonal directions. Thus, a protein grid is scanned in seven directions with a step of 1Å. If a scanning line experiences a protein-solvent-protein (PSP) situation during the scanning, the voxels contained in the intermediate solvent are marked as the PSP voxels. Each grid voxel value is increased by one when it undergoes a PSP event in a direction. This means that the minimum value of a voxel is 0 (the voxel has not experienced any PSP events in any direction) and the maximum value of a voxel is 7 (the voxel experienced PSP events in all seven directions). Thus, the higher value of a voxel corresponds to a higher probability of it belonging to a cavity. The representation of the improved LIGSITE method is shown in Fig. 8.

#### Van der waals potential (channel 2)

Van der Waals force is a common intermolecular force and an important factor for the binding of a protein and a molecule. Detailed analysis of the van der Waals force field around a protein can improve the accuracy of prediction of the pocket position. The construction of this grid channel uses the ideas of the energy-based methods. The probe is placed at various grid positions; then, the van der Waals forces between the protein and the probe are calculated to obtain the van der Waals force energy distribution. The probe used here is −*CH*_3_, which is a functional group commonly found in the drug molecules. The 12-6 Lennard-Jones equation[21] was used to calculate the van der Waals potential energy:

1 $$ E_{VDW}=\sum\limits_{i,j}^{}\left(\frac{A}{r^{12}}-\frac{B}{r^{6}}\right)  $$

where:

2 $$ A=\epsilon r_{0}^{12}  $$

3 $$ B=2\epsilon r_{0}^{6}  $$

**Fig. 9:**
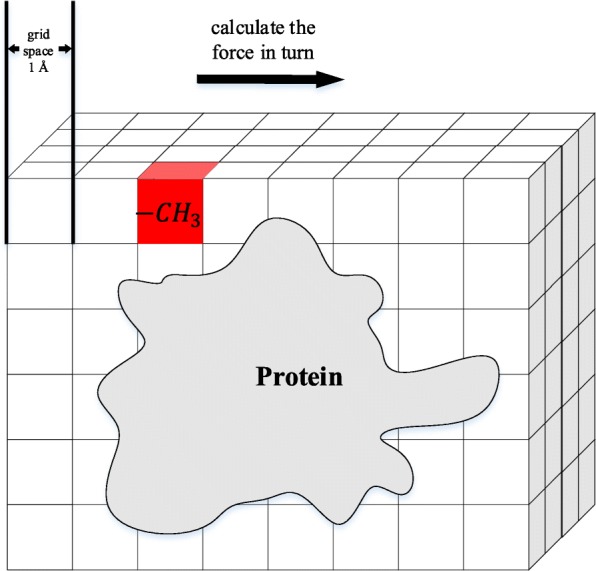
The calculation process of the van der Waals force channel grid. The probe is placed in each grid voxel in turn, and the van der Waals potential between the probe and the protein is calculated as the voxel value

Here, *i* and *j* are the atoms of −*CH*_3_ and protein, respectively, *ε* is the depth of the potential well and *r*_0_ is the distance when the potential reaches its minimum. To calculate the potential for two particles, including atom *i* and atom *j*, the same procedure as Amber in Autodock [40] is used, which sets $\epsilon =\sqrt {\epsilon _{i} \epsilon _{j}}$, *r*_0_=*r*_*i*_+*r*_*j*_. Finally, the sum of the potential *voxel*_*value*=*E*_*VDW*_ between the probe atoms and protein atoms is set as the value of the grid voxel when the probe is placed at this grid. The process is shown in Fig. 9.

The calculation of the potential of the entire grid of proteins is a time-consuming process. To alleviate this computation pressure, a cut-off radius of 8Å was set. When the distance between a pair of atoms is over 8Å, their force will be ignored. The cut-off radius setting was also applied in the following two channels.

#### Hydrogen bond potential (channel 3)

Hydrogen bonding is a non-bonded interaction stronger than the van der Waals force; it cannot be ignored when a molecule binds to a protein. Here, we used an approach similar to that used in the case of the van der Waals potential (channel 2) and calculated the hydrogen bond potential with the hydrogen (−*OH*) probe. The hydrogen atom can act as a hydrogen bond acceptor and a donor, and is a common functional group in drug molecules. To calculate the hydrogen bond potential, the 12-10 Lennard-Jones equation was used, similar to the ff86 force field in Amber [41]:

4 $$ E(i,j)=\left(\frac{C}{r^{12}}-\frac{D}{r^{10}}\right)  $$

where:

5 $$ C=5\epsilon r_{0}^{12}  $$

6 $$ D=6\epsilon r_{0}^{10}  $$

The parameters atom *i* and atom *j* belong to the probe and protein, respectively, which are the atoms that may form the hydrogen bonds. The parameters *ε* and *r*_0_ are the same parameters as the Amber filed parameters in Autodock. For example, a well depth of 5 kcal/mol at 1.9Å with oxygen was used. The *r* value is the distance between atom *i* and atom *j*. In addition, because of the saturation of the hydrogen bond, the sum of the hydrogen bond energy of the probe and all potential atoms of the protein are no longer used. Instead, the value with the maximum absolute value is introduced as *E*_*HBond*_ and is calculated as follows.

7 $$ (\tilde{i},\tilde{j})=\mathop{\arg\max}_{i,j}\left|E(i,j)\right|  $$

8 $$ E_{HBond}=E(\tilde{i},\tilde{j})  $$

#### Electric potential energy (channel 4)

Coulomb force plays an important role in the formation of the binding sites. The analogue of channel 2 and 3 was used to calculate this potential energy grid. The probe selected here is no longer a specific functional group but is a particle with single positive charge that is placed in each grid to calculate the corresponding voxel value. It should be emphasized that calculation of the Coulomb force between a positively charged particle and a protein requires information on the partial charges of each atom of the protein, which is described in the pdbqt file. The equation for calculation of the Coulomb force is shown below.

9 $$ E_{electric}=\sum\limits_{e,j}^{}K\frac{q_{1}q_{2}}{r^{2}}  $$

*K* is the Coulomb constant, particle *e* is the unit positive charge particle with the charge *q*_1_ of + 1, atom *j* is an atom of the protein with partial charge *q*_2_, and *r* is the distance between the particle *e* and the atom *j*.

### Training

**Fig. 10:**
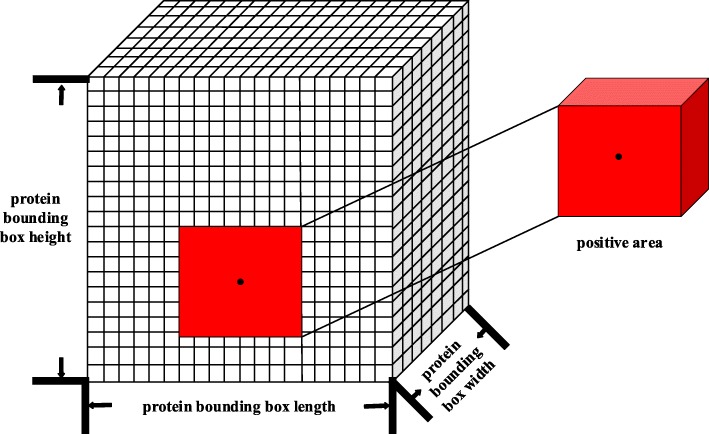
Determination of the positive samples. The black dot is the geometric center of the protein binding site, and a square (red block in the figure) with a side length of 20Å centered on it is set as the positive sample area; the total may include 4×4×4=64 sampling blocks, which are marked as positive samples

After the four-channel grid descriptor was obtained, a 16Å ×16Å ×16Å block sampling was implemented. The sample blocks within 2Å of the center of the site were set as positive samples because the 2Å setting ensures that each protein produces enough positive samples (64 sampling blocks) for the training while maintaining accuracy; the 2Å setting will produce an area with a side length of 20Å (16Å + 2Å + 2Å = 20Å). The process is shown in Fig. 10.

**Fig. 11:**
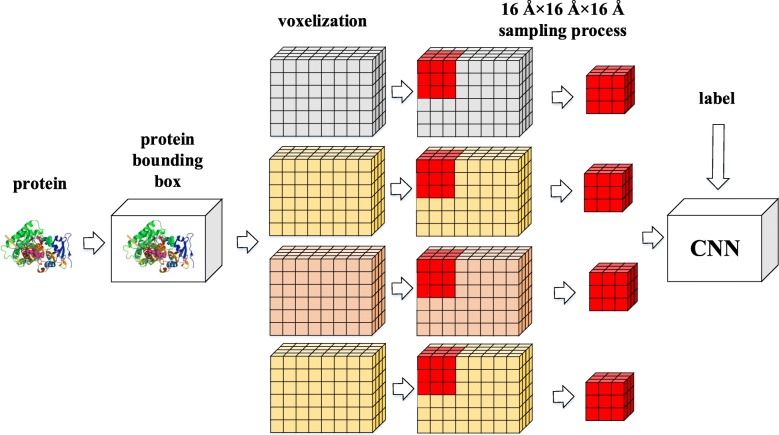
Training flow chart (4 channels)

After obtaining the positive samples, the protein bounding box was sampled by 16Å ×16Å ×16Å block in steps of 4Å; if a sample box is not within the binding site area, it is marked as a negative sample. Finally, to reduce the serious imbalance of the sample ratios, the negative samples were sampled down to 64 samples; finally 128 sample blocks were used for each protein. The sampling process is shown in Fig. 11.

The deep learning framework was implemented using Keras [42]. The neural network built here has a simpler and deeper architecture compared with that of DeepSite; the details are shown in Table 4.

**Table 4:** Neural network architecture

layer number	structure of each layer	kernel size	layer input	layer output
1	Conv3D,ReLU	2,(8, 8, 8)	4,(16,16,16)	2,(16,16,16)
2	Conv3D,ReLU	4,(8, 8, 8)	2,(16,16,16)	4,(16,16,16)
3	MaxPooling3D	(2,2,2)	4,(16,16,16)	4,(8,8,8)
4	Dropout(0.25)	NA	4,(8,8,8)	4,(8,8,8)
5	Conv3D,ReLU	8,(4, 4, 4)	4,(8,8,8)	8,(8,8,8)
6	Conv3D,ReLU	16,(4, 4, 4)	8,(8,8,8)	16,(8,8,8)
7	MaxPooling3D	(2,2,2)	16,(8,8,8)	16,(4,4,4)
8	Conv3D,ReLU	32,(2, 2, 2)	16,(4,4,4)	32,(4,4,4)
9	Conv3D,ReLU	64,(2, 2, 2)	32,(4,4,4)	64,(4,4,4)
10	Dropout(0.25)	NA	32,(4,4,4)	64,(4,4,4)
11	Flatten	NA	32,(4,4,4)	64,(4,4,4)
12	Dense(128),ReLU	NA	4096	128
13	Dropout(0.5)	NA	128	128
14	Dense(1),sigmoid	NA	128	1

It should be noted that the calculated voxel values of each channel (surface-solvent-surface events, hydrogen bond potential energy, van der Waals potential energy, electric potential energy) are in different ranges and thus need to be normalized before training. However, large number of sampling blocks was obtained. After the experiments, we found that the mapping of the arctangent function had a good normalization performance and enabled quick convergence of the model weights. All voxel values can be mapped to (-1, 1) according to the equation:

10 $$ voxel\_value=\frac{2}{\pi}\arctan(voxel\_value)  $$

### Prediction

Once the model is obtained, it can be used to predict the binding sites of a new protein. In the prediction process, the sampling step size is set to 4Å, and finally, the predicted value of each 16Å ×16Å ×16Å block of the protein is obtained; the value corresponds to the probability that each sampling block belongs to a site. Clustering analysis of the prediction results is required to predict multiple binding sites. The Density-Based Spatial Clustering of Applications with Noise (DBSCAN) clustering method is used, which can divide the area into the clusters with sufficiently high density and can find clusters of arbitrary shapes in the space of noise [39]. The final predictions are obtained by dividing all sample blocks into various classes. The DBSCAN algorithm requires two parameters, *Eps* (epsilon, the maximum distance between two samples to be considered in the same neighborhood.) and *MinPts* (the minimum number of points required to form a dense region). In the experiment, we set *Eps*=*step*_*size*+1 thus increasing the sampling step size by 1. The performance of various *Minpts* values was compared in the experiment.

The output of the model is the probability that a sample block belongs to the binding site; the output value ranges from 0 to 1. Therefore, it is necessary to set a threshold to indicate whether a sample is positive. Here, the threshold is set to 0.5, because the majority of the value of the positive predictions are close to 1 and the values of the negative predictions are close to 0. After the threshold screening and DBSCAN clustering, the sample blocks can be divided into multiple potential binding site regions, and the geometric center of all sampling blocks in the same cluster is calculated as the center of the pocket. At the same time, each pocket is scored based on the average predicted block probability values of each pocket (sampling blocks that are clustered to a single class) calculated as the score of a pocket.

## Additional files

Additional file 1 Protein list for the experiment with various channels. This file contains randomly selected training proteins for the experiment with various channels. All proteins come from the sc-PDB database, 3000 for training, 1000 for validation and 1000 for testing. (CSV 81 kb)

Additional file 2 Protein list for the experiment with various DBSCAN parameters. This file contains randomly selected training proteins for the experiment with various DBSCAN parameters. All proteins come from the sc-PDB database, 3000 for training, 1000 for validation and 1000 for testing. (CSV 81 kb)

Additional file 3 Protein list for the experiment on sc-PDB using various methods. This file contains randomly selected proteins for the experiment with various methods. They are used in the 5-fold cross-validation experiments. A total of 5000 proteins were randomly selected from the sc-PDB database are used in the experiment, with an average of 1000 proteins per fold. (CSV 68.4 kb)

Additional file 4 Protein list for the experiment on PDBbind. This file contains randomly selected proteins for the experiment on PDBbind. A total of 5000 proteins were used including 3000 proteins from the sc-PDB database constitute the training set, 1000 proteins from the sc-PDB database constitute the validation set, 1000 proteins from the PDBbind database constitute the test set. All proteins are randomly selected, and the three sets of proteins do not intercept. (CSV 79.1 kb)

## Data Availability

All data used in the experiments are from public databases, including sc-PDB (2017) and PDBbind (2018). The detailed lists of proteins used in the experiment are included in the additional files. The source code is available at https://github.com/595693085/ProteinDescriptor.

## Abbreviations

DBSCAN: Density-based spatial clustering of applications with noise
Eps: Epsilon, the maximum distance between two samples for them to be considered as being in the same neighborhood
EBMC: Efficient Bayesian multivariate classifier
L-J: Lennard-Jones
LR: Logistic regression
MinPts: The minimum number of points required to form a dense region
PAM: Point accepted mutation
PCP: Physicochemical property
PSP: Protein-solvent-protein
RGB: Red, green and blue
SVM: Support vector machine
